# Azacitidine and decitabine show distinct response patterns in pediatric Myelodysplastic Syndromes and Juvenile Myelomonocytic Leukemia before HSCT: a systematic review and quantitative analysis

**DOI:** 10.1186/s13148-026-02191-0

**Published:** 2026-07-01

**Authors:** Alessandra Cianflone, Francesco Cecere, Luigi Coppola, Fabiana Cacace, Peppino Mirabelli, Francesco Paolo Tambaro

**Affiliations:** 1 Clinical and Translational Research Unit, AORN Santobono Pausilipon-IRCCS, Naples, Italy; 2 Stem Cell Transplantation and Cell Therapy Unit, AORN Santobono Pausilipon-IRCCS, Naples, Italy

**Keywords:** Pediatric Hematologic Malignancies, Hematopoietic Stem Cell Transplantation, Hypomethylating Agents, Azacitidine, Decitabine, Myelodysplastic Syndromes, Juvenile Myelomonocytic Leukemia, Epigenetic Dysregulation

## Abstract

**Background:**

Epigenetic dysregulation is a key therapeutic target in pediatric hematologic malignancies resistant to conventional chemotherapy, where allogeneic hematopoietic stem cell transplantation (HSCT) remains the only curative option. In this setting, hypomethylating agents (HMAs), such as azacitidine (AZA) and decitabine (DAC), are increasingly used as pre-transplant bridging strategies to reduce disease burden and enhance treatment sensitivity. Their integration with HSCT may improve pre-transplant remission rates, prolong complete remission, and increase the likelihood of durable disease control and cure. However, evidence in pediatric patients remains limited. Here, we performed a systematic review and pooled analysis to evaluate the efficacy of pre-HSCT epigenetic therapy in pediatric hematologic malignancies.

**Results:**

A pooled analysis of seven studies of 87 pediatric patients including 51 Myelodysplastic Syndromes (MDS) and 36 Juvenile Myelomonocytic Leukemia (JMML) treated with DAC or AZA prior to HSCT was performed. Statistical analyses used chi-squared or Fisher’s exact tests for categorical variables and non-parametric tests for continuous variables, with correction for multiple comparisons. In MDS, Complete remission (CR) was achieved in 64.7% of patients and strongly associated with DAC, translating into improved post-HSCT survival compared to PD (84.2% vs. 42.8%). In JMML, partial remission (PR) and progressive disease (PD) predominated (47.2% and 41.7%, respectively), while CR was rare (11.1%); response was not associated with HMA type or genetic features, although older age correlated with PD. Comparative analysis confirmed superior responses in MDS (higher CR and SD) versus JMML, which showed higher PR and PD rates. PTPN11 mutations were significantly associated with multiple clinical variables, including treatment response.

**Conclusions:**

Pre-transplant HMA therapy shows variable efficacy in pediatric myeloid malignancies. DAC induces rapid, deep responses in MDS, improving post-HSCT survival. JMML responds less, yet post-transplant survival remains favorable. Higher HMA responsiveness in MDS likely reflects predominant epigenetic dysregulation, whereas JMML is mainly driven by aberrant RAS/MAPK pathway activation. These results emphasize that pre-transplant strategies should be tailored to the specific biological and clinical context of each disease. However, their impact relative to upfront HSCT or conventional chemotherapy remains to be definitively determined.

**Graphical abstract:**

**Figure:**
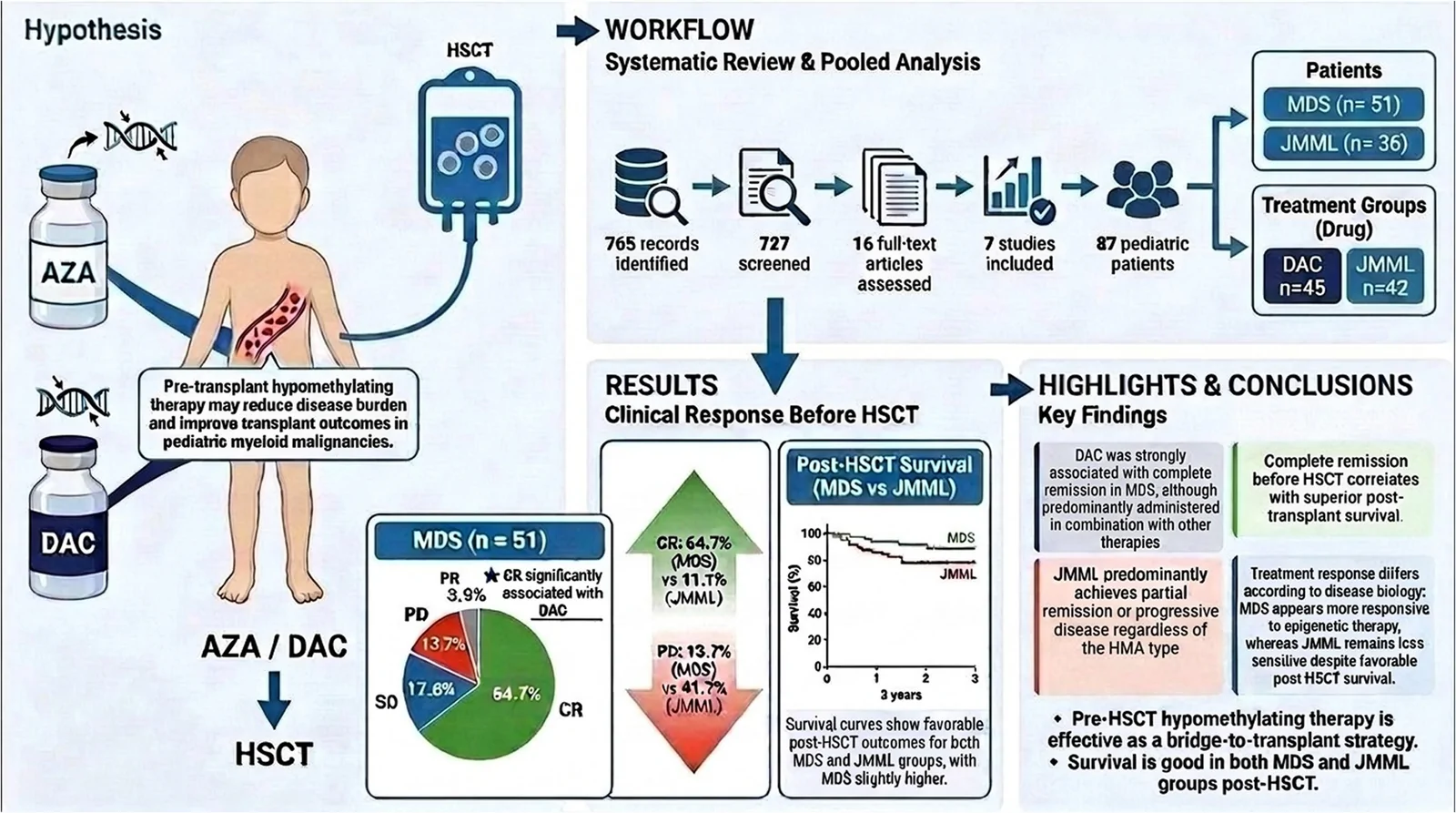

**Supplementary Information:**

The online version contains supplementary material available at https://doi.org/10.1186/s13148-026-02191-0.

## Background

Epigenetic dysregulation plays a key role in the pathogenesis of hematologic malignancies, particularly in myeloid neoplasms [1]. Disruption of epigenetic regulation provides a target for anti-cancer therapies, including HMAs and histone deacetylase inhibitors (HDACi), which, by inhibiting DNA methyltransferase or histone deacetylase activity, promote DNA hypomethylation or histone acetylation, thereby restoring the expression of silenced tumor suppressor genes or promoting gene transcription [2, 3]. To date, in the pediatric population, only AZA, a DNA demethylating agent (DNMTi), has been approved by the FDA for the treatment of Juvenile Myelomonocytic Leukemia (JMML) [4]. Other epigenetic drugs (e.g., decitabine, vorinostat, romidepsin, panobinostat, tazemetostat, IDH inhibitors) are currently approved mainly for adult malignancies or are under clinical investigation in pediatric populations, and still lack an FDA pediatric indication for hematologic diseases [5–8]. Allo-HSCT remains the only curative strategy for pediatric hematologic malignancies with poor response to conventional chemotherapy, particularly in myeloid diseases. Combinations of epigenetic drugs with conventional chemotherapy and allo-HSCT are currently under clinical investigation, aiming to increase sensitivity to anti-cancer therapies and improve patient outcomes. In adults with high-risk Myelodisplastic Syndrome (hrMDS) or Acute Myeloid Leukemia (AML), hypomethylating agents such as AZA or DAC are used both pre-transplant as a cytoreductive “bridge” [9] and post-transplant as maintenance, the latter being generally safe and associated with favorable clinical outcomes in terms of relapse rates and disease-/overall-free survival [10–12]. In pediatric patients, data are more limited. Prospective trials of epigenetic priming with DAC prior to induction chemotherapy in AML have demonstrated feasibility [13], although definitive transplant-specific endpoints have not yet been established. Given the increasing clinical interest in epigenetic therapies as pre-transplant treatment, a systematic evaluation of available data is needed. On this basis, in this study we performed a systematic review and data analysis of published studies investigating the use of epigenetic drugs prior to HSCT in pediatric patients affected by hematologic malignancies.

## Methods

The paper was reported in accordance with the Preferred Reporting Items for Systematic Reviews (PRISMA) guidelines [14]. All data originate from previously published papers in international peer-reviewed journals. The PRISMA checklist is shown in Supplementary Table 1. The review was not registered and no protocol was prepared.

### Outcome measures

The primary aim was to evaluate treatment response of pre-HSCT epigenetic therapy, alone or in combination, in pediatric hematologic malignancies. Secondary objectives included assessing treatment efficacy including overall and event free survival, relation to patient demographics (age, sex), disease characteristics (bone marrow blast percentage at diagnosis, germiline mutations, karyotype), treatment variables (number of cycles, response assessment).

### Literature search

Relevant studies were identified through electronic searches of PubMed, Embase, Web of Science, Cochrane Library, and ClinicalTrials.gov, with the last search performed on 14 October 2025 using combined title and abstract terms: (allogeneic OR allo-HSCT OR hematopoietic stem cell transplantation OR bone marrow transplant) AND (leukemia OR acute lymphoblastic OR ALL OR acute myeloid OR AML OR MDS OR myelodysplastic syndrome) AND (epigenetic OR hypomethylating agent OR azacitidine OR decitabine OR HDAC inhibitor OR vorinostat OR panobinostat OR tazemetostat OR DNMT inhibitors OR HDAC inhibitors OR EZH2 inhibitors OR hypomethylating agents OR epigenetic therapy) AND ( child* OR pediatric OR paediatric OR adolescent OR infant). The search was limited to English-language articles. Clinicaltrial.gov was screened by the following terms combinations: *Condition or Disease*: leukemia OR acute lymphoblastic OR ALL OR acute myeloid OR AML OR myelodysplastic syndrome OR MDS; *Other terms*: epigenetic OR hypomethylating agent OR azacitidine OR decitabine OR HDAC inhibitor OR vorinostat OR panobinostat OR tazemetostat OR DNMT inhibitors OR HDAC inhibitors OR EZH2 inhibitors OR hypomethylating agents OR epigenetic therapy; *Treatment*: allogeneic OR allo-HSCT OR hematopoietic stem cell transplantation OR bone marrow transplant.

### Study selection

Studies on pre-HSCT epigenetic therapy in pediatric hematologic disorders were screened and assessed by independent reviewers (AC, FC, PM, LC), with full texts evaluated and disagreements resolved by consensus (FPT, AC).

### Eligibility criteria and data abstraction

Included studies comprised clinical trials and observational studies, including comparative, randomized, multicenter, and validation designs. Excluded were case reports, reviews, non-hematologic studies, preclinical or in vitro research, adult-only or non-human studies, and trials without reported results. Extracted data included study details, patient demographics, disease characteristics, epigenetic therapy regimens, concomitant treatments, treatment response outcomes, genetic and cytogenetic features, blast percentage at diagnosis, treatment cycles, cycle of response assessment, follow-up from HSCT and survival status.

### Risk of bias assessment

The methodological quality of the included non-randomized studies was assessed using the MINORS criteria [15].

### Data analysis

To improve the robustness of the analysis, patients with missing data for the primary outcome (Clinical Response) were excluded from outcome-based analyses. Associations between categorical variables (sex, HMA treatment, blast category, karyotype and survival status) and Clinical Response were assessed using the chi-squared test or, when any expected count fell below 5, Fisher’s exact test with Monte Carlo simulation (10,000 replicates). Pairwise comparisons between Clinical Response groups were performed using Fisher’s exact test. Differences in continuous variables across Clinical Response categories were evaluated using the non-parametric Kruskal–Wallis test. For overall comparisons, p-values derived from the same family of analyses were adjusted for multiple testing using the Benjamini–Hochberg false discovery rate (FDR) method. For pairwise post hoc tests, Bonferroni correction was applied within each variable. Statistical significance after correction was defined as q < 0.05. All analyses were conducted using R (version ≥ 4.0). Data were imported using the readxl package (v1.4.5) [16], and plots were generated with ggplot2 (v4.0.1) [17]. Statistical tests and post hoc analyses were performed using the stats (v4.5.2) and rstatix (v0.7.3) packages [18]. Statistical significance after correction was defined as q < 0.05, in particular *p* < 0.0001 (****), *p* < 0.001 (***), *p* < 0.01 (**), *p* < 0.05 (*) and p > = 0.05 (n.s.).

## Results

### Search results

**Fig. 1:**
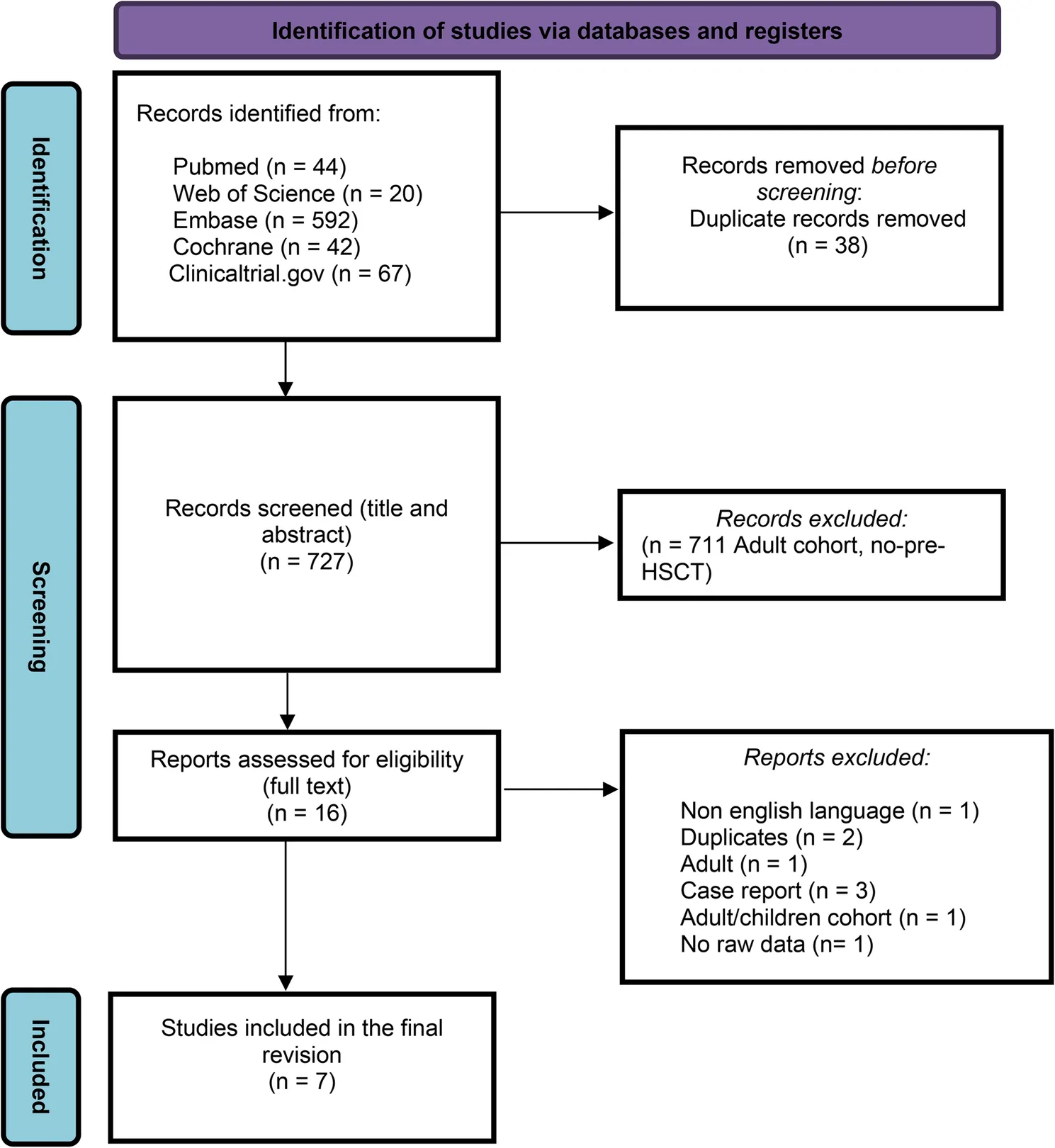
PRISMA flowchart of the selected studies

A total of 765 records were identified through database searches. After removing 38 duplicates, 727 records were screened by title and abstract, of which 711 were excluded for not meeting inclusion criteria (e.g., adult populations, non-epigenetic pre-HSCT therapies, reviews, case reports, preclinical studies, off-topic). Sixteen full-text articles were assessed for eligibility, and nine were excluded (case reports (*n* = 3), adult population (*n* = 1), non-English publication (*n* = 1), duplicate (*n* = 2), mixed adult/pediatric population (*n* = 1), reporting aggregate data only (*n* = 1)). Seven studies met the inclusion criteria and were included in the qualitative synthesis. The selection process is shown in Fig. 1.

### Features of the included studies

Seven studies assessed treatment response to AZA (*n* = 4 studies) [4, 19–21] and to DAC (*n* = 3 studies) [22–24] before HSCT in MDS and JMML, with three including comparison/control groups; two were phase II multicenter AZA trials (NCT02447666).

### Quality assessment

The methodological quality of the included studies was overall moderate. Comparative studies (*n* = 3) achieved scores of 14–18, showing adequate baseline comparability and statistical analyses but lacking sample size calculation and blinded endpoint assessment. Non-comparative studies (*n* = 4) scored 8–13, mainly due to absence of sample size planning, and unblinded outcome evaluation. Most studies were retrospective (5/7), contributing to these limitations, whereas the two phase II trials demonstrated higher methodological rigor. Detailed MINORS scores are reported in Supplementary File 2.

### Diagnostic criteria

Included patients were diagnosed between November 2007 and March 2023 according to WHO 2008, WHO 2016, and WHO 2022/ICC classifications [25–27] including pediatric-adapted WHO criteria [28]. Bone marrow blast thresholds for MDS remained largely consistent across WHO classifications, categorized into three ranges: <5%, 5–9%, and 10–19%, while ≥ 20% blasts are classified as AML in the later classifications. Across classification systems, ring sideroblast cut-offs for SF3B1-mutated cases were retained, and molecular alterations were incorporated in the most recent classifications. MDS subtypes have evolved across WHO classifications. WHO 2008 included entities such as Refractory Cytopenia with Unilineage Dysplasia (RCUD), Refractory Cytopenia of Childhood (RCC, pediatric), and Refractory Anemia with Excess Blasts (RAEB) [25]; while WHO 2016 refined these into MDS with Single Lineage Dysplasia (MDS-SLD), MDS with Multilineage Dysplasia (MDS-MLD), MDS with Ring Sideroblasts (MDS-RS, including SF3B1-mutated cases), MDS with Excess Blasts (MDS-EB), MDS with isolated del(5q), and Unclassifiable (MDS-U), retaining RCC as a pediatric entity and building on earlier revisions that replaced FAB categories such as RAEB and RAEB-T [29]. The CCC system classifies pediatric MDS into Refractory Cytopenia (RC, corresponding to WHO RCC) and Refractory Cytopenia with Excess Blasts (RCEB), which broadly corresponds to advanced MDS with increased blasts (5–19%) [28]. Despite changes in nomenclature, core diagnostic criteria, including dysplasia and clinical features, have remained largely stable. More recent classifications (WHO 2022 and ICC) have further refined disease subtypes and increasingly incorporated genetic alterations into disease definition [30]. For the purpose of this study, pediatric MDS cases were stratified by bone marrow blast percentage into RCC (< 5%), advanced MDS (5–19%), and MDS/AML (20–29%); however, according to WHO 2016/2022, ≥ 20% blasts defines AML, and the term “MDS-AML” is used here for reporting purposes only. JMML diagnostic criteria have remained largely stable across EWOG-MDS and Children’s Oncology Group definitions, with WHO 2016 formally incorporating RAS pathway mutations (PTPN11, NRAS, KRAS, NF1, and CBL) as key diagnostic markers [25, 26].

### Clinical response evaluation criteria

Treatment response across studies was assessed using standardized categories: Complete Remission (CR), Partial Remission (PR), Stable Disease (SD) and Progressive Disease (PD) according to modified International Working Group (IWG) criteria for pediatric MDS [31] and international JMML response criteria [32]. Response to pre-transplant HMA treatment was analyzed in the overall cohort, separately in MDS and JMML patients, and comparatively between the two groups. All data used for the analyses are reported in Supplementary File 3.

### Disease Subtypes of the Analyzed Cohort

Of 127 initially included patients (87 MDS, 40 JMML), MDS cases were aMDS (*n* = 56) and MDS-AML (*n* = 24), with few low-grade (RCC, *n* = 7) and rare secondary MDS (sMDS) (*n* = 2). JMML patients were typically < 3 years old, with RAS/MAPK mutations, monocytosis, leukocytosis, thrombocytopenia, < 20% blasts, and frequent spleno/hepatomegaly. Patients lacking pre-HSCT response data (*n* = 28) were excluded. Additionally, patients not receiving epidrug therapies (*n* = 12) were excluded due to insufficient sample size after stratification by disease and treatment. In total, 87 patients were included in the final analysis.

### Pre-transplant treatments

Forty-two patients received AZA and 45 DAC. AZA was administered either IV or SC at 50–100 mg/m²/day for 5–7 days every 28–42 days (most commonly 75 mg/m²/day × 7 days; 2.5 mg/kg/day in infants < 10 kg), for a median of 3 cycles (range: 1–11). DAC monotherapy was administred at 20 mg/m²/day IV for 5 consecutive days every 4 weeks (typically 3–4 cycles), with some patients having received prior low-dose cytarabine (50–100 mg/m²/day) or etoposide (50 mg/m²/day) for 3–5 days. DAC-based combinations included: DAC 20 mg/m²/day IV (days 1–5) plus cytarabine 10 mg/m² every 12 h (days 6–15) with mitoxantrone 5 mg/m² (days 6, 8, 10) (*n* = 9), homoharringtonine 1 mg/m² (days 6–12) (*n* = 2), or idarubicin 5 mg/m² (days 6–8) (*n* = 2); G-CSF (5 µg/m²/day, days 6–15). was included in all treatment. No other treatments were administered before HMA therapy. Study characteristics are summarized in Table 1.

**Table 1:** Clinical, demographic and therapeutic characteristics of the included studies.

	Doi	Population	Size	Age (Median [range], y)	Sex (%, m)	Monotherapy (*N*°)	Association (*N*°)	NT (*N*°)	Only CHT (*N*°)	HMA Dosage (mg/m^2^)/ Dose Schedule	Cycle treatment (median, range)	OS (%) /y	Follow-up (median, [range], m)
1	(19) Phase 2, open-label	aMDS	10	13.3 [1.9–15.9]	60	10	-	-	-	AZA/ 75/ daily1-7 every 28-days	3 (1–3)	70.0/ 5.3	44.4 [5.7 − 86.9]
		MDS-AML	-										
2	(23) Retrospective	JMML	10	2.88 [0.5–4.25]	90	5	5	-	-	DAC/ 20/daily 1–5 every 28-days cycle	3 (1–5)	84.8/ 3.0	26.0 [4.0–49.0]
3	(22) Retrospective	aMDS	15	6.6 [1.6–11.5]	60.7	3	13	8	4	DAC/ 20/n.a.	2 (1–4)	71.4/ 4.0	53.0 [2.3–127.0]
		MDS-AML	6										
		RCC	7										
4	(20) Retrospective / letter	JMML	12	4.8 [0.4–9.1]	66.6	10	2	-	-	AZA/50–100/ daily 1–5 every 28-days or daily 5–7 every 28–42 days cycle	5.5 (1–11)	n.a.	16.0 [1.0–66.0]
5	(21) Retrospective	aMDS	20	10.85 [6-17.2]	36.3	8	-	11	3	AZA/ 75/ daily 1–7 every 28-days cycle	3 (1–13)	100/ 4.0	32.0 [7.0-54.4]
		MDS-AML	2										
6	(4) phase 2, open-label	JMML	18	2.1 [0.3–6.9]	61.1	18	-	-	-	AZA/ 50–100/ Daily 1–7 every 28-days	3 (3–6)	90.0/ 3.6	23.0 [7.0-39.3]
7	(24) Retrospective	aMDS	11	8.0 [2.0-15.0]	63	3	17	2	5	DAC/ 20/ daily 1–5; n.a.	n.a.	84.8/3.0	26.0 [4.0–49.0 ]
		MDS-AML	16										

*DOI* Digital Object Identifier, *aMDS* Advanced Myelodysplastic Syndrome, *JMML* Juvenile Myelomonocytic Leukemia *RCC* Refractory Cytopenia of Childhood, *AZA* Azacitidine, *DAC* Decitabine, *NT* Not-Treated, *CHT* Chemotherapay, *OS* Overall Survival, *y* Years, *m* Months, *n.a* Not Available

### Clinical and demographic patient characteristics

Across the cohort of 87 patients, the median age was 5 years (range: 0.3–17.2); 66.7% were male. The median treatment cycle count was 3 (range: 1–11) and response assessed after a median of 3 cycles (range: 1–3). Median post-HSCT interval, defined as the time period between HSCT and the assessment of clinical response, was 3.03 years (range: 0.08–10.58). Survival status at last follow-up was available for 65 patients. Of these, 76.9%% (*n* = 50) were alive, while 23.0% (*n* = 15) were deceased. Deaths occurred due to relapse (*n* = 4); accidental death (*n* = 1); transplant-related mortality (*n* = 6). Missing data were primarily observed in treatment-related variables. Demographics, molecular markers, and clinical response data were complete across all patients. Missing data for each out come are reported in Supplementary File 3.

### MDS cohort clinical and demographic characteristics

Fifty-one MDS patients were analyzed, *n* = 40 aMDS (78.4%), *n* = 9 MDS-AML (17.6%), and *n* = 2 sMDS (3.9%). Males accounted for 62.5% of aMDS and 66.7% of MDS-AML. The two sMDS were female. Median age was 7.5 years for aMDS (range: 1.3–13.6), 8.5 years for MDS-AML (range: 1.6–17.2), 6.5 years for sMDS aged 4 and 9 respectively. Median blasts was of 10.5% (range: 6.0–15.25) and 22.0% (range: 20.0–23.0) for aMDS and MDS-AML, respectively, data for the sMDS group were not available. All patients received a median of 2 treatment cycles (range: 1–3), with response assessed after 1.5 cycles (aMDS) and 2 cycles (MDS-AML); data for the sMDS group were not available. The median follow-up from HSCT was 3.85 years (range: 0.29–10.58) for aMDS and 3.94 years (range: 0.48–8.79) for MDS-AML. Data for the sMDS patients were not available. Germline mutations were assessed in 42.6% of the overall cohort, with missing data exceeding 50.0% due to lack of investigation in the original studies. In the aMDS subgroup, GATA2 and SETBP1 were the most frequently mutated genes (10.0%, *n* = 4 each), followed by NRAS and PTPN11 (7.5%, *n* = 3 each), and CBL, ETV6, FLT3/ITD, and RUNX1 (5.0%, *n* = 2 each); all other genes were each mutated in a single patient within the cohort. In the sMDS subgroup, RUNX1, SAMD9, TP53, and ZRSR2 mutations were each identified in one patient. MDS-AML group showed no mutations. Cytogenetics was avaible for all patients; 48.9% (*n* = 23) of patients had a normal karyotype, while monosomy 7 (-7) was observed in 21.3% (*n* = 10), trisomy 8 in 6.4% (*n* = 3), and complex karyotype in 4.3% (*n* = 2); other abnormalities each accounted for 2.1% (*n* = 1) of the whole cohort. Overall, 82.8% of patients were alive at the last follow-up. Given the comparable distribution of variables between aMDS and MDS-AML, correlation analyses were conducted by merging these subgroups into a single MDS category. Blast percentage was analyzed separately in relation to treatment response. Due to the small sample size and substantial missing data, patients with sMDS (*n* = 2) were also included in the overall MDS category. Table 2 showed details of demographic and clinical data.

### JMML cohort clinical and demographic characteristics

Thirty-six JMML patients were included. Males comprised 75.0% of the cohort. Median age was 2.82 years (range: 0.3–6.9 years), and median blast percentage at diagnosis was 6.0% (range: 1.0–19.0%). Patients received a median of 4 treatment cycles (range: 1–11), with the third cycle serving as the reference timepoint for clinical response assessment. Median follow-up from HSCT was 1.8 years (range: 0.8–5.5 years), however this variable was characterized by a high proportion of missing data (75.0%). Overall, 72.2% of patients remained alive at the time of the last follow-up. Mutational analysis covered the 100% of included subjects and showed that PTPN11 was the most frequently altered gene, detected in 66.7% (*n* = 24) of patients. NRAS mutations were observed in 19.4% (*n* = 7), followed by NF1 in 13.9% (*n* = 5) and KRAS in 11.1% (*n* = 4). JAK3 mutations were less common, occurring in 5.6% (*n* = 2) of cases. No other mutations were identified. Karyotype covered the 72.3% of total JMML cohort. Analysis showed that 60.7% (*n* = 17) of patients had a normal karyotype, while monosomy 7 was observed in 25% (*n* = 7) of cases. Other abnormalities, including -Y, + 21, del [5](q13q33), and del [9](q12q21), each occurred in one patient. Overall, 72.2% of patients were alive at the last follow-up. Table 2 showed details of demographic and clinical variables.

**Table 2:** Patient Profiles in MDS and JMML Cohorts. N, sample size; Q1, 25th percentile; Q3, 75th percentile; SD, Standard Deviation.

MDS	*N*	Median	Q1	Q3	Mean	SD
Age (Y)	51	8.0	5.33	11.0	8.1	3.72
BM Blasts at Diagnosis (%)	29	15.0	8.0	20.0	14.64	6.74
Treatment Cycles (N°)	22	2	1	3	2.05	0.84
Response Assessment (N° cycle)	29	2	1	3	1.86	0.88
Post HSCT interval (Y)	29	3.33	1.93	5.88	3.87	2.79
JMML						
Age (Y)	36	2.82	1.17	4	2.79	1.83
BM Blasts at Diagnosis (%)	18	6.0	2.25	8.75	6.5	5.04
Treatment Cycles (N°)	18	4	3	6.75	4.72	2.65
Response Assessment (N° cycle)	25	3	3	3	3	0
Post HSCT interval (Y)	9	1.08	0.42	3.17	1.7	1.86

### Treatment response associated to HMAs

**Fig. 2:**
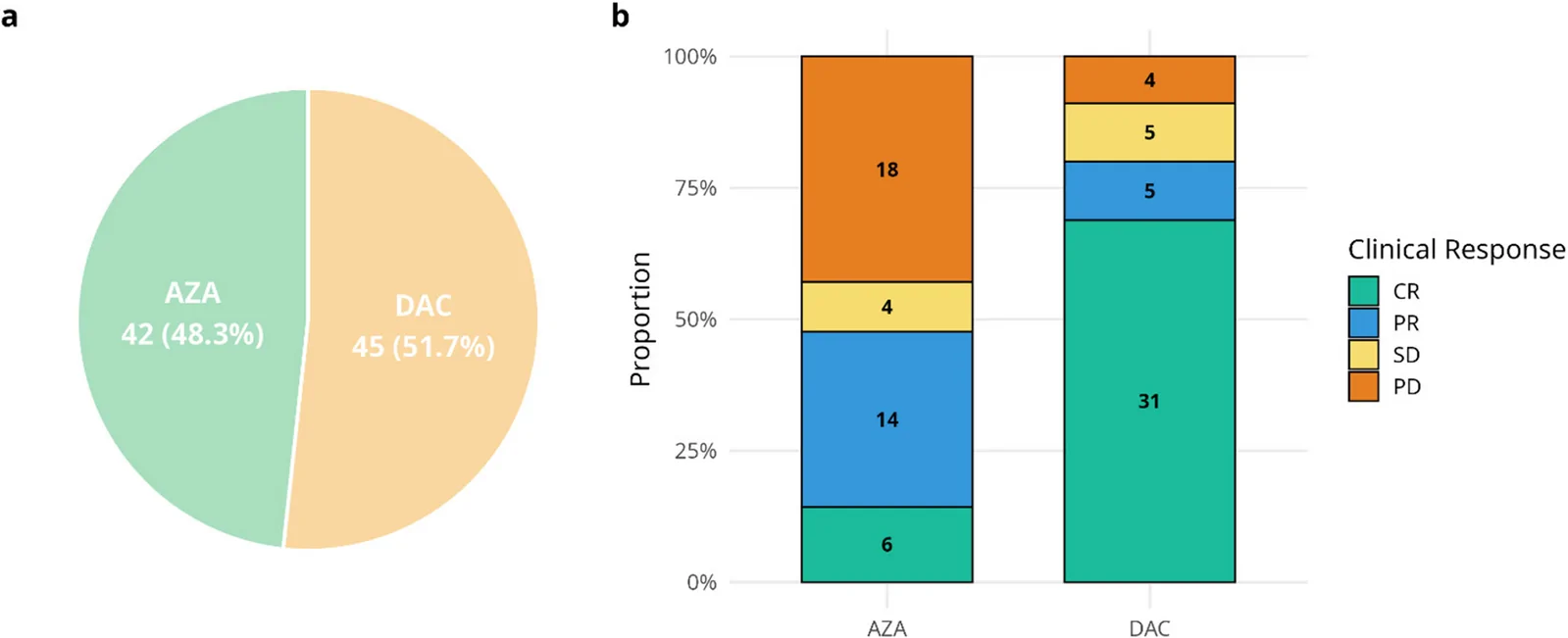
Clinical Response distribution in AZA and DAC treated patients. 2**a;** Overall clinical response categories distribution; 2**b;** HMA treatment distribution; 2**c; **Distribution of clinical responses in relation to DAC and AZA treatment

Overall, 51.7% of patients received DAC and 48.3% received AZA (Fig. 2a); AZA was admnistered as monotherapy, whereas DAC was given alone in 13.2% (*n* = 5) and in combination with chemotherapy in the other 82.2% of cases. Clinical response was significantly associated with epidrug type (*p* < 0.0001): CR was more frequently observed in DAC-treated patients (*p* < 0.0000001), whereas PD was more common among AZA-treated patients (*p* < 0.0001); PR was slightly more frequent with AZA (*p* < 0.05) (Fig. 2b). A total of 72.2% of patients were alive at last follow up. Sex, blast percentage at diagnosis, number of treatment cycles, follow-up, and survival status were not significantly associated with response. No statistically significant association was found between karyotype and clinical outcomes. Interstingly, based on mutational status, PTPN11 was the only gene significantly associated with clinical response (*p* < 0.0001), age (*p* < 0.000001), blast percentage (*p* < 0.001), number of treatment cycles (*p* < 0.0001), and cycle at response assessment (*p* < 0.0001).

### Treatment response in MDS cohort

**Fig. 3:**
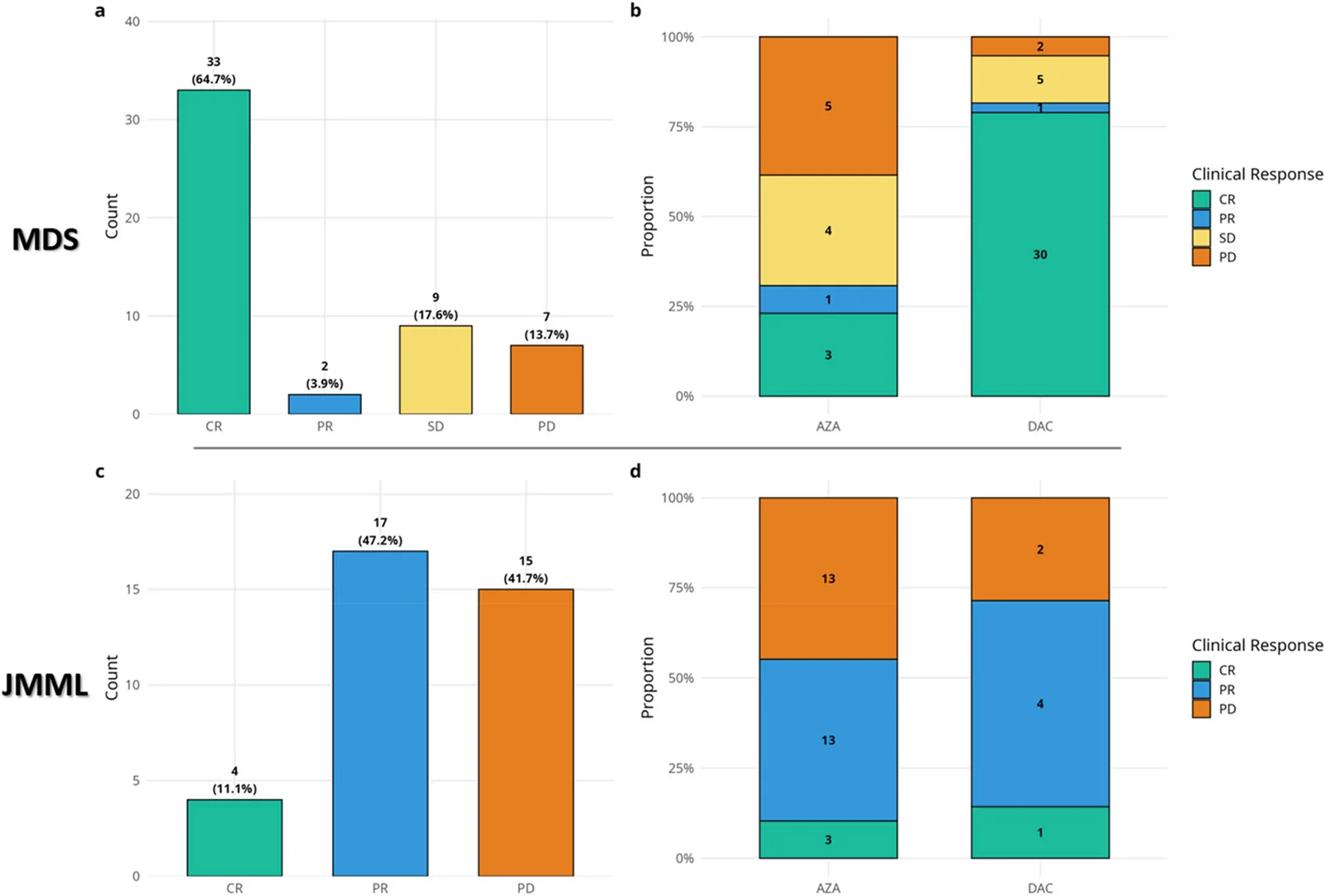
Clinical response distribution in MDS and JMML. 3**a;** Proportion of treatment response in MDS; 3**b;** Proportion of treatment response in MDS stratified for HMAs; 3**c;** Proportion of treatment response in JMML; 3**d;** Proportion of treatment response in JMML stratified for HMAs

DAC was administered to 74.5% of MDS patients (*n* = 32 aMDS, 6 MDS-AML, 2 sMDS, ) and AZA to 25.5% (*n* = 13), with DAC given as monotherapy in 13.2% of aMDS cases (*n* = 5). Given the small number of patients receiving decitabine monotherapy, the correlation with response was performed in the overall cohort without stratifying patients into monotherapy and combination therapy groups. Of all treatment responses, CR was the most frequent (64.7%, *n* = 33), followed by SD (17.6%, *n* = 9), PD (13.7%, *n* = 7), and PR (3.9%, *n* = 2) (Fig. 3a). Treatment response was significantly associated with epidrug type (*p* < 0.0001), with CR more frequent in DAC-treated patients (*p* < 0.0001) and PD in AZA-treated patients (*p* < 0.001) (Fig. 3b). Among the five patients treated with monotherapy, two achieved SD and three achieved CR. Given the potential association of GATA2 and RUNX1 alterations with germline predisposition syndromes, treatment responses were further examined in these patients. Among the four patients harboring GATA2 mutations, two achieved CR, one had SD, and one experienced PD. The two patients with SD and PD had received DEC, whereas the two patients achieving CR were treated with AZA and DEC, respectively. All three patients with RUNX1 mutations received DEC; two achieved CR and one achieved PR. A total of 84.2% of patients achieving CR were alive at a median follow-up of 4.4 years (*p* < 0.001) compared with 42.8% at 5.5 years in patients with PD (data not shown). All patients in SD and PR remained alive at 2.49 and 0.58 years post-HSCT, respectively (data not shown). No significant associations were observed for sex, age, blast percentage at diagnosis, number of treatment cycles, or mutation profile. No statistically significant association was found between karyotype and clinical outcomes.

### Treatment response stratified by blast percentage in MDS subgroups

**Fig. 4:**
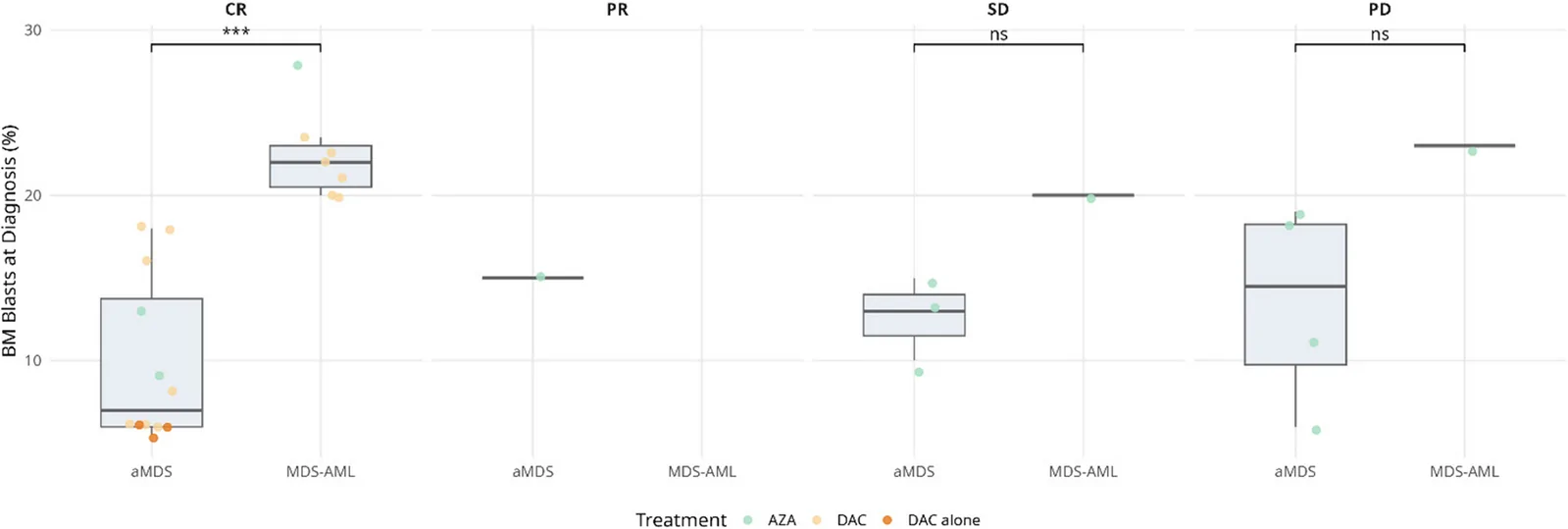
Distribution of clinical responses according to bone marrow blasts percentage in AZA and DAC treated aMDS and MDS-AML groups

When MDS was divided into two subgroups on the basis of blasts percentage at diagnosis (aMDS: 5.0–19.0%; MDS-AML: 20.0–29.0%), analysis showed that, irrespective of blasts percentage range, CR was more observed in DAC treatment while PD, PR and SD in AZA treatment. Pairwise analysis showed no significant differences across clinical response categories (Fig. 4).

### Treatment response in JMML cohort

Across the JMML cohort, 80.6% (*n* = 29) of patients were treated with AZA and 19.4% (*n* = 7) with DAC. DAC was given as monotherapy to 42.9% ( *n* = 3 patients). PR was the most frequent clinical outcome (47.2%, *n* = 17), followed by PD (41.7%, *n* = 15) and CR (11.1%, *n* = 4). No cases of SD were registered (Fig. 3c). Treatment response was not significantly associated with the type of epidrug (Fig. 3d). Age was correlated with clinical response, with PD patients having a higher median age compared to PR and CR (median age: PD: 4.0 years, range 1.1–6.9; PR: 2.04 years, range 0.3–5.4; CR: 0.8 years, range 0.1–2.83), particularly PR median age was significantly lower than PD (*p* < 0.05). No other significant associations were observed for sex, blast percentage at diagnosis, post HSCT interval, survival status at last follow-up, number of treatment cycles, or the treatment cycle at which response was assessed. Gene mutations were not significantly associated with treatment response or other variables. Similarly, no statistically significant association was found between karyotype and clinical outcomes.

### Comparison between MDS and JMML

**Fig. 5:**
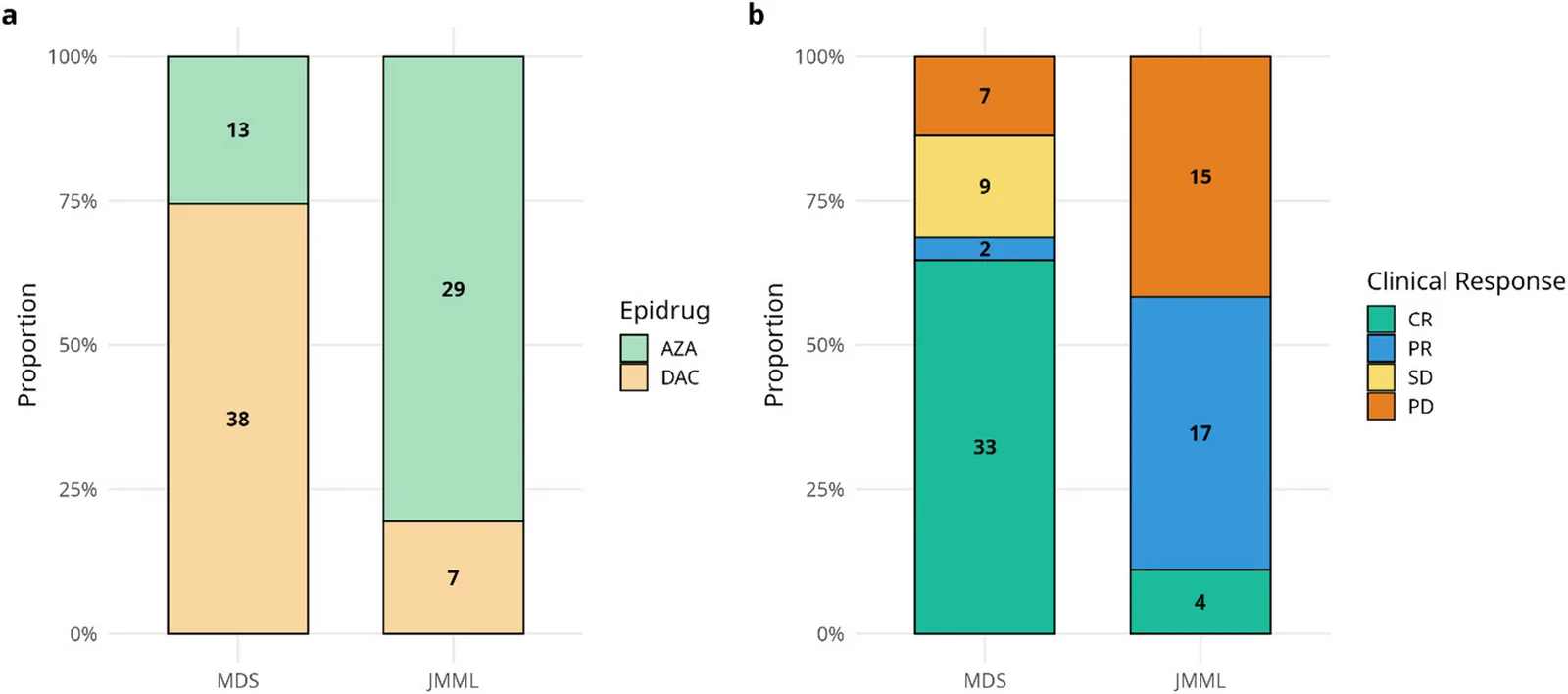
HMA and clinical response proportion in MDS and JMML

**Fig. 6:**
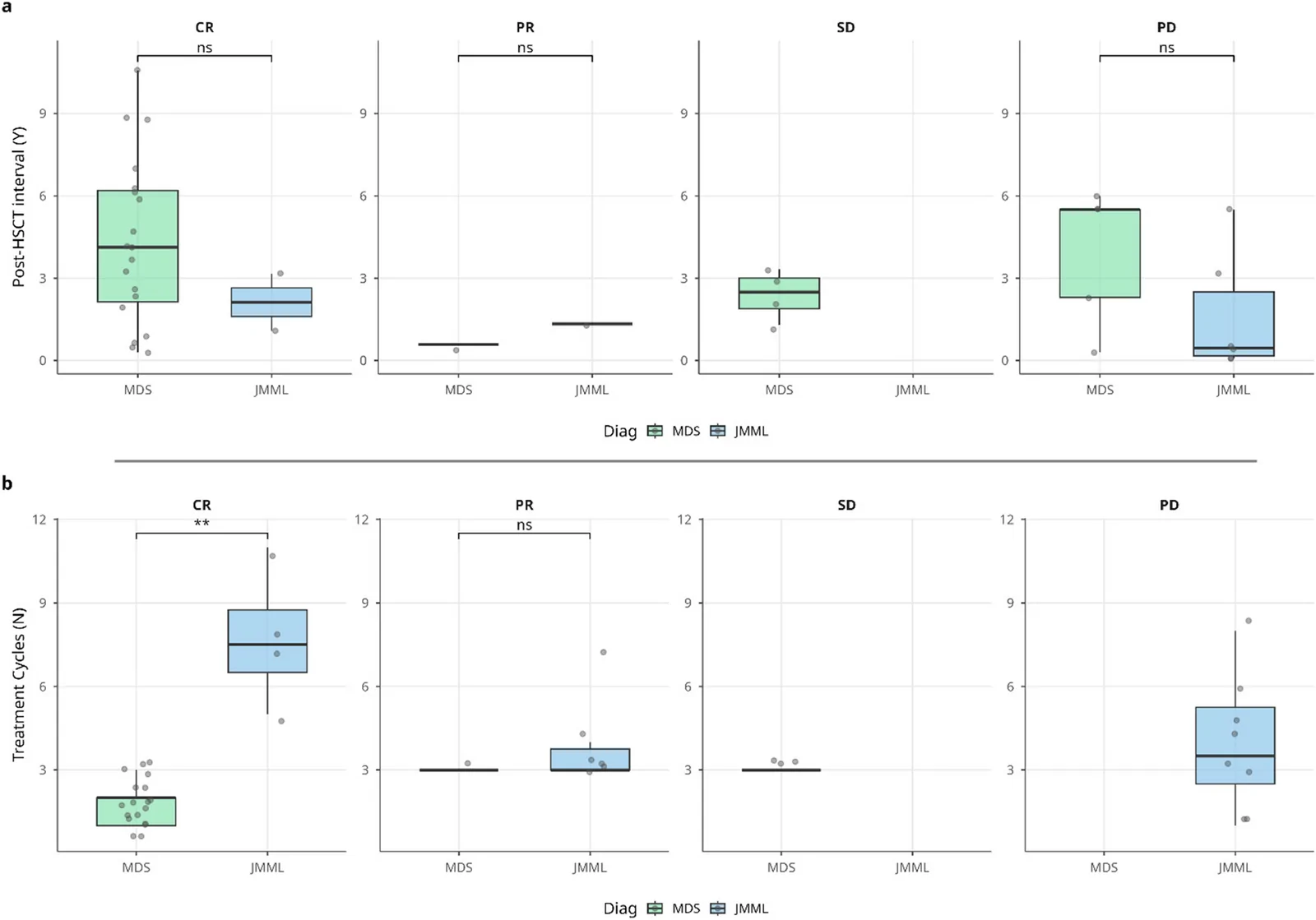
Comparison between post HSCT interval (**a**) and number of treatment cycles (**b**) between MDS and JMML in relation to treatment response

The two groups differed in age, percentage of blasts at diagnosis, number or treatment cycle, clinical response assessment cycle, follow-up (supplementary File 4) and treatment (Fig. 5a). With regards to demographics, the two groups were comparable in terms of sex distribution; however, as expected, age was significantly higher in the MDS group. The high percentage of missing data for blasts percentage in JMML cohort (55.0%) limited a comparative analysis in respect to this variable. With respect to treatment response, a significantly higher proportion of CR was observed in the MDS cohort compared to the JMML cohort (64.7% vs. 11.1%, *p* < 0.0001), whereas PR were more frequent in JMML than in MDS (47.2% vs. 3.9%, *p* < 0.000001). In terms of disease control, SD was more common in MDS compared with JMML (17.6% vs. 0%, *p* < 0.001), whereas PD occurred more frequently in JMML than in MDS (41.7% vs. 13.7%, *p* < 0.001) (Fig. 5b; Table 3). A longer follow up was for the MDS group compared to JMML, however no significant value were observed between post HSCT interval and clinical responses categories (Fig. 6a). Number of treatment cycle were higher in JMML in respect to MDS, particularly for CR category (*p* < 0.001, Fig. 6b), however JMML group presented a high percentage of missing data for this variable (50.0%). Sex, use of DAC as monotherapy, and survival status at the last follow-up were not significantly associated with clinical response category.

**Table 3:** Different distribution of clinical response categories in MDS and JMML.

MDS	*N*	%	JMML	*N*	%
SD	9	17.6	**PR**	17	47.2
PR	2	3.9	**PD**	15	41.7
PD	7	13.7	**CR**	4	11.1
CR	33	64.7	**SD**	–	–
***COMPARATIVE ANALYSIS***					
***Category***	***Higher in***	***p value***	***Sig***	***MDS (%)***	***JMML (%)***
SD	MDS	9.12e-03	***	17.6	0
PR	JMML	1.64e-06	****	3.9	47.2
PD	JMML	5.27e-03	***	13.7	41.7
CR	MDS	6.07e-07	****	64.7	11.1

## Discussion

Epigenetic dysregulation represents a central mechanism in the pathogenesis of myeloid malignancies, particularly when HSCT represents the only curative treatment [33–35]. Given this context, HMA therapies are being explored as a bridging strategy before transplantation in MDS and JMML, with the aim of improving clinical response by simultaneously targeting epigenetic dysregulation and restoring bone marrow functionality. In our study, the analysis of treatment responses in pediatric MDS and JMML before HSCT highlights marked differences between the two diseases. In the MDS cohort, clinical outcomes were significantly influenced by the choice of hypomethylating agent. DAC was the predominant therapy and was associated with high complete response rates, while progression occurred less frequently. Moreover, DAC demonstrated marked efficacy in rapidly reducing blast burden in MDS, often achieving complete remission within the first two treatment cycles. In contrast, JMML patients primarily received AZA, and treatment responses were dominated by partial responses and progressive disease, with complete responses being uncommon. Unlike MDS, response in JMML showed no clear association with the type of hypomethylating agent, suggesting that disease biology rather than therapy choice may play a stronger role in determining outcomes. Comparative analysis confirmed these data, underscoring that MDS patients exhibit higher rates of complete response when treated with DAC, whereas JMML AZA-treated patients more frequently experience partial response or disease progression. These differences likely reflect the distinct biological characteristics and treatment sensitivities of the two disorders. JMML and MDS are not directly comparable; therefore, differences in response rates between the two should be interpreted within this context. However, in JMML, even partial responses achieved prior to HSCT have been associated with improved post-transplant progression-free survival [36], highlighting that attainment of complete remission is not always necessary nor achievable given the disease’s intrinsic resistance to conventional therapies. Nevertheless, long-term survival following HSCT was relatively favorable in both groups, likely reflecting the practice of proceeding to transplant promptly after therapy. Therefore, although direct comparison between MDS and JMML has inherent limitations, these findings highlight the importance of tailoring therapeutic strategies to the specific disease biology and clinical context. From a mechanistic point of view, although AZA and DAC are both cytidine analogues that act as hypomethylating agents, they exhibit important differences that may explain their variable activity across diseases characterized by distinct pathogenic mechanisms. AZA is primarily incorporated into RNA and, to a lesser extent, into DNA, thereby affecting both protein synthesis and epigenetic regulation. In contrast, DAC is incorporated almost exclusively into DNA, resulting in a more direct and potent inhibition of DNA methylation [37, 38]. These pharmacological differences may partially account for the distinct therapeutic responses observed in different hematologic malignancies. In MDS, epigenetic dysregulation plays a central role in disease pathogenesis, with aberrant DNA methylation leading to the silencing of genes that can be reactivated through hypomethylating therapy [39]. Consequently, agents that directly target DNA methylation may be particularly effective in this context. By contrast, JMML is predominantly driven by activating mutations in components of the RAS/MAPK signaling pathway, such as PTPN11 and NF1. These mutations promote increased proliferation and survival of leukemic clones, mechanisms that are less directly influenced by epigenetic modulation [40–42]. As a result, JMML cells may exhibit reduced sensitivity to hypomethylating agents when compared with diseases in which epigenetic alterations represent a primary pathogenic driver. This is reflected in our cohort by the requirement of a higher number of HMA cycles to achieve CR in JMML. Experimental studies further support that RAS-driven JMML cells resist DAC-induced apoptosis and may rely on persistent MAPK signaling for survival, providing a mechanistic explanation for these differential responses [42, 43]. From a regulatory and clinical perspective, AZA is approved the by the FDA for JMML, and several clinical trials have been conducted or are ongoing (NCT05849662). In contrast, DAC is not currently approved for pediatric use, and no relevant clinical trials are underway to evaluate its efficacy in pediatric hematologic diseases. These differences underscore not only regulatory considerations but also highlight the limited clinical experience and evidence base for DAC in children, which may partly explain its underutilization despite potential mechanistic rationale. Data from our analysis underlie that DAC is predominantly explored in MDS, reflecting its established use in adult high‑risk MDS since its approval by FDA in 2006 and its role as an epigenetic priming agent [44], while the broader investigation of AZA in JMML, particularly in protocols aimed at bridging to HSCT, likely reflects differences in disease biology and clinical need [45]. Importantly, the MDS cohort of the AZA-JMML-001 trial, the only prospective study investigating hypomethylating agents in pediatric MDS, showed no confirmed responses after three treatment cycles and was therefore terminated early, highlighting the limited prospective evidence currently available in this setting. Although direct head‑to‑head pediatric comparisons are lacking, adult studies suggest similar overall efficacy for both agents, with AZA sometimes associated with higher overall response and survival, while DAC may achieve comparable complete remission rates [46]. Mechanistic differences may further inform investigator choice in pediatric trials [47]. Together, these factors help explain the observed pattern of DAC in MDS-focused studies versus AZA in JMML protocols. The main strength of our study lies in the high homogeneity of data and clinical features among included subjects, including diagnosis, treatment schedule, and dosage. Nevertheless, several limitations restrict the interpretation of results. Overall, the reduced sample size after stratification and the predominance of retrospective data limit the statistical power of the analyses. From an interpretative standpoint, several additional limitations should be considered. Notably, none of the included studies provide a direct comparison between patients undergoing upfront HSCT and those receiving HMAs prior to transplantation, in both MDS and JMML. In MDS cohorts, AZA was mainly used as a bridging strategy to HSCT in treatment-naïve patients, without a comparator group undergoing immediate transplantation or alternative cytotoxic chemotherapy, thus precluding any meaningful assessment of survival differences between approaches. Similarly, in JMML, both prospective and retrospective studies evaluating AZA as pre-transplant therapy lack a control group treated with upfront HSCT, limiting conclusions on the relative benefit of hypomethylating agents in terms of overall survival (OS) and leukemia-free survival (LFS) [4, 20]. Another relevant aspect concerns the use of DAC which is more often administered in combination rather than as monotherapy. The only available comparative data address different HMA-based strategies: specifically, no significant differences in long-term outcomes were observed between DAC alone and DAC-based combination regimens, with comparable 5-year OS and LFS rates. In contrast, pre-transplant response consistently emerges as a major prognostic factor. Patients achieving clinical complete or partial remission show significantly superior outcomes compared with those with stable or progressive disease, with 5-year OS of 96.2% versus 72.2% and LFS of 95.5% versus 48.9%, respectively (*p* < 0.001) [48].Taken together, these findings suggest that, in the absence of randomized comparisons, the depth of response prior to HSCT may be more relevant than the specific pre-transplant strategy itself, while the role of HMAs relative to upfront HSCT or conventional chemotherapy remains to be clearly defined.

## Conclusion

In conclusion, hypomethylating agents can play a valuable role as a bridge to HSCT in pediatric myeloid malignancies, but their efficacy is highly disease-dependent. DAC demonstrated substantial activity in MDS, inducing rapid blast reduction and high rates of complete response, which translated into prolonged post-HSCT survival. By contrast, JMML showed more limited sensitivity to HMAs, with responses dominated by partial remission or progressive disease, reflecting the predominant role of RAS/MAPK pathway mutations rather than epigenetic dysregulation. These findings underscore that while HMAs can effectively reduce disease burden and potentially improve transplant outcomes in MDS, their utility in JMML may be constrained by underlying disease biology, highlighting the need for tailored pre-transplant strategies and combination approaches to better optimize efficacy. However, while HMAs are effective bridge to HSCT, the absence of direct comparisons with upfront HSCT or conventional chemotherapy limits conclusions on their relative benefit.

## Supplementary Information

Supplementary Material 1.

Supplementary Material 2.

Supplementary Material 3.

Supplementary Material 4.

## Data Availability

All data supporting the findings of this study are available within the paper and its Supplementary Information.
