# Supplementary material for: Azacitidine and decitabine show distinct response patterns in pediatric Myelodysplastic Syndromes and Juvenile Myelomonocytic Leukemia before HSCT: a systematic review and quantitative analysis
Source: Clin Epigenetics. 2026 Jul 1;18:169. doi: 10.1186/s13148-026-02191-0 (PMC13595803; doi:10.1186/s13148-026-02191-0)
Supplement: Supplementary file 2 [file 13148_2026_2191_MOESM2_ESM.docx]

|  | | | **F. Locatelli et al.2023** | **Y.Cai et al.2023** | **J. Gao et al. 2022** | **A. Cseh et al. 2015** | **N. Waespe et al.2016** | **C.M. Niemeyer et al.2021** | **Y. Ren et al. 2024** |
| --- | --- | --- | --- | --- | --- | --- | --- | --- | --- |
|  | **ITEMS** | |  |  |  |  |  |  |  |
|  | 1 | Clearly Stated Aim | 2 | 2 | 1 | 1 | 1 | 2 | 2 |
|  | 2 | Inclusion of consecutive patients | 2 | 1 | 1 | 1 | 2 | 2 | 1 |
|  | 3 | Prospective Collection of Data | 2 | 2 | 2 | 1 | 2 | 2 | 2 |
|  | 4 | End Points Appropriate to Study Aim | 2 | 2 | 2 | 2 | 2 | 2 | 2 |
|  | 5 | Unbiased Assessment of Study End Point | 1 | 0 | 0 | 0 | 0 | 0 | 0 |
|  | 6 | Follow-Up Period Appropriate to Study Aim | 2 | 1 | 2 | 1 | 2 | 2 | 2 |
|  | 7 | Less than 5% Lost to Follow Up | 2 | 2 | 2 | 2 | 2 | 2 | 2 |
|  | 8 | Prospective Calculation of the Study Size | 0 | 0 | 0 | 0 | 0 | 0 | 0 |
| **COMPARATIVE STUDIES**  **COMPARATIVE STUDIES** | 9 | Adequate Control Group | n.a. | n.a. | 1 | n.a. | 2 | n.a. | 1 |
|  | 10 | Contemporary Groups | n.a. | n.a. | 2 | n.a. | 1 | n.a. | 0 |
|  | 11 | Baseline Equivalence of Groups | n.a. | n.a. | 2 | n.a. | 2 | n.a. | 2 |
|  | 12 | Adequate Statistical Analysis | n.a. | n.a. | 2 | n.a. | 2 | n.a. | n.a |
|  |  | Total score | 13 | 11 | 17 | 8 | 18 | 12 | 14 |

**Supplementary file 2.** Methodological quality assessment of the 7 included studies using MINORS score. Items were scored 0–2; “n.a.” indicates not applicable for non-comparative studies.
